# Dietary Patterns and Association with Anemia in Children Aged 9–16 Years in Guangzhou, China: A Cross-Sectional Study

**DOI:** 10.3390/nu15194133

**Published:** 2023-09-25

**Authors:** Jie Ma, Jie Huang, Chunzi Zeng, Xuexin Zhong, Weiwei Zhang, Bo Zhang, Yan Li

**Affiliations:** 1 School of Public Health, Southern Medical University, Guangzhou 510515, China; jasmine225@smu.edu.cn (J.M.); zhangbo2018@smu.edu.cn (B.Z.); 2 Department of Foodborne Diseases and Food Safety Risk Surveillance, Guangzhou Center for Disease Control and Prevention, Guangzhou 510440, China; huangjie1026@126.com (J.H.); gzcdc_zengcz@gz.gov.cn (C.Z.); gzcdczhangww@foxmail.com (W.Z.); 3 School of Public Health, Sun Yat-Sen University, Guangzhou 510080, China; zhongxx27@mail.sysu.edu.cn

**Keywords:** children, dietary pattern, anemia, Cantonese cuisine, China

## Abstract

Anemia affects 1.8 billion people worldwide, and diet is one of the key modifiable factors for treating anemia in children. The dietary pattern has changed rapidly in recent decades, but its effect on childhood anemia has not been reported. This study aimed to identify dietary patterns among children in rural areas of Guangzhou, China, and explore their association with anemia. A total of 1476 children aged 9–16 years old were included in this study. Demographics, lifestyle, and anthropometric and dietary information were collected. Factor analysis was used to identify dietary patterns, and anemia was diagnosed based on hemoglobin levels. Robust Poisson regression and subgroup analysis were used to analyze the association between dietary patterns and anemia. The prevalence of anemia in children was 10.4%, with 6.1% in boys and 15.4% in girls. Four dietary patterns were identified, including a fast food pattern, a vegetarian pattern, a meat and egg pattern, and a rice and wheat pattern. A high score in the fast food pattern was positively associated with risk of anemia in children entering puberty (PR = 1.767, 95% CI: 1.026~3.043, *p* = 0.039), especially in girls after menarche, with marginal significance (PR = 1.740, 95% CI: 0.977~3.097, *p* = 0.059). A high score in the meat and egg pattern was negatively associated with risk of anemia in children entering puberty (PR = 0.498, 95% CI: 0.286~0.866, *p* = 0.013), especially in boys after spermatorrhea (PR = 0.237, 95% CI: 0.031~0.590, *p* = 0.007). The fast food pattern was a risk factor for anemia in children, and the meat and egg pattern was a protective factor for anemia in children entering puberty. The findings of this study could be used to guide the strategies of evidence-based preventive nutrition interventions to curb anemia in children.

## 1. Introduction

Anemia remains a major public health problem, affecting one-fifth of the world’s population, with the global number of people with anemia increasing from 1.4 billion in 1990 to 1.8 billion in 2019 [1]. According to the 2020 Chinese Residents’ Nutrition and Health Survey, the anemia rate among children aged 6~17 was 6.1% [2], whereas the anemia rate among children aged 6~17 in areas covered by the “Nutrition Improvement Plan for Rural Compulsory Education Students” in 2019 was 8.7%. Overall, the anemia rate in rural areas is significantly higher than the national average [3], and there is still a lot of room for improvement in the anemia situation among rural children nationwide. We need to pay more attention to the prevalence of anemia among rural children.

Childhood is a critical period for growth and reproductive maturity. The demand for nutrients increases during this period, making adolescents more susceptible to nutrient deficiencies [4,5], which may lead to anemia. Anemia in children can have widespread negative effects throughout the lifespan, such as reduced resistance, impaired physical performance, and impaired neurological development [6,7]. Therefore, it is essential to pay timely attention to and improve the anemia status of children.

Risk factors for anemia include diet, genetic factors, pregnancy, and some pathological factors, and diet is one of the main controllable factors. The overall dietary situation is presented by dietary patterns, which refer to the types, quantities, and proportions of various foods in daily diet [8]. Compared with studies focusing on single nutrients or foods, dietary pattern analysis comprehensively considers the potential interactions between nutrients and foods, presenting cumulative effects, and is helpful in formulating nutritional interventions [8,9]. Dietary pattern analysis has been widely used to investigate the relationship between diet and chronic diseases.

A study in Mexico found that the Western dietary pattern (mainly characterized by sweet drinks, salty snacks, charcuterie, fast food, and cereals/tubers) was positively associated with anemia in girls aged between 12 and 19 years [10]. A study in Japan found that a dietary pattern including vegetables, condiments, mushrooms, and red meat was negatively correlated with anemia in elderly individuals aged ≥ 65 years [11]. Although there have been studies investigating the association between dietary patterns and anemia, few articles specifically highlight the characteristics of the dietary patterns of Cantonese cuisine in South China, which is a classic example of Eastern healthy dietary patterns. Guangzhou, an important city in southern China near Hong Kong, is famous for its traditional Cantonese cuisine. Therefore, this study aims to explore the association between dietary patterns and anemia among rural children in Guangzhou.

## 2. Materials and Methods

### 2.1. Participants

This is a cross-sectional study that was conducted from April 2021 to June 2022. A multi-stage stratified cluster random sampling method was used to recruit study participants: (1) Three primary schools and three secondary schools were randomly selected from rural areas of Guangzhou. (2) A stratified sampling method was used to select three grades from each primary school and two grades from each secondary school. (3) Two to three classes of students were randomly selected from each grade.

**Figure 1:**
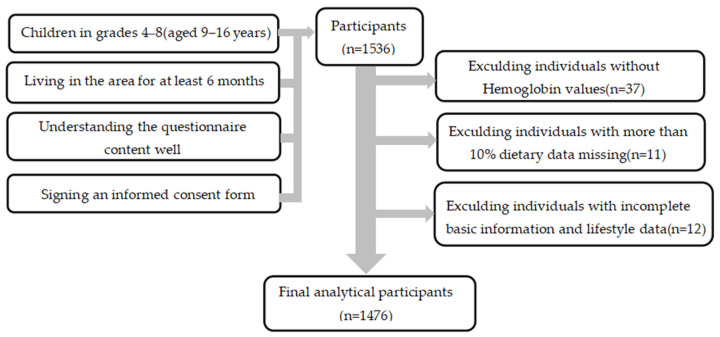
Flowchart of the selection of research participants.

The sample size was calculated using the following formula: $N=\mathrm{deff}\frac{u_{\alpha /2}^{2}P\left(1-P\right)}{\delta^{2}}$ [12]. The meanings and values of various parameters were as follows: 95% confidence level and $u_{\alpha /2}$ = 1.96, corresponding to a probability P of 7% based on the 2016 detection rate of anemia among compulsory education children in Guangzhou [13]; the design effect (deff) was defined as 1.5; the relative error (r) was set as 25%; and δ was equal to 25% × 7%. The calculated sample size was 1225 students. Considering invalid questionnaires and refusal rates, the actual sample size was expanded by 10%, and the survey required at least 1347 students. The screening process for survey respondents is shown in Figure 1.

### 2.2. Survey Content

Children were interviewed face to face by a uniformly trained research assistant in order to complete the survey. The survey content included questionnaire surveys, physical examinations, and laboratory tests.

(1) Questionnaire survey: (1) Demographic information included age, gender, region, place of residence, parents’ education level, etc. (2) Lifestyle variables included smoking, drinking, moderate-to-high physical activity, physical education classes, sedentary time, bedtime, and sleep time. (3) Dietary surveys were conducted using a semi-quantitative food frequency questionnaire (FFQ) to investigate the frequency and intake of food consumed by students in the past month. Food models and pictorials aided participants in assessing their food intake. Based on the food frequency questionnaire derived from the China National Chronic Non-communicable Disease and Nutrition Surveillance in 2015 [14] and the dietary characteristics of children in Guangzhou, the questionnaire was adjusted by a panel of experts, including scientists in the fields of epidemiology and nutrition. In the FFQ, 66 types of food across 19 categories were included based on the Chinese Food Composition Table Standard Edition (6th edition) [15,16].
(2) Physical examination: Height and weight were measured by a mechanical height meter and an electronic scale, respectively, with a measurement accuracy of 0.1 cm and 0.1 kg, respectively. All examination instruments and methods were in accordance with the Chinese national standard of anthropometric measurement methods in health surveillance [17]. Body mass index (BMI) was calculated as BMI = weight (kg)/height (m^2^). The criteria for assessing the nutritional status were determined by the 2007 WHO BMI-for-age reference (age was calculated as the date of investigation minus the date of birth) [18], including four categories: BMI Z score < −2 indicates malnutrition, −2 ≤ BMI Z score < 1 indicates normal, 1< BMI Z score ≤ 2 indicates overweight, and BMI Z score > 2 indicates obesity. BMI Z scores were calculated by WHO AnthroPlus 3.0 software. Based on the WS/T 456-2014 “Screening of Malnutrition in School-Age Children and Adolescents” [19], children with growth retardation were considered those whose height was less than or equal to the height threshold for their age group.
(3) Laboratory examination: Hemoglobin levels were measured with the cyanmethemoglobin method. According to the World Health Organization’s 2011 “Haemoglobin concentrations for the diagnosis of anemia and assessment of severity’” [20], anemia was diagnosed with the following criteria: Children aged 5–11 years with Hb < 115 g/L were diagnosed with anemia, those aged 12–14 years with Hb < 120 g/L were diagnosed with anemia, males aged 15 years and above with Hb < 130 g/L were diagnosed with anemia, and non-pregnant females aged 15 years and above with Hb < 120 g/L were diagnosed with anemia.

### 2.3. Dietary Pattern Establishment

For the exploratory factor analysis of dietary patterns, 66 food items were grouped into 19 predetermined food categories, as shown in Table 1. The correlation matrix between the 19 food groups was statistically tested, with the Kaiser–Meyer–Olkin (KMO) test yielding > 0.8 and the Bartlett’s sphericity test yielding a significant *p* (*p* < 0.001), indicating that the correlation between variables was sufficient for factor analysis. Principal component analysis was used to determine the common factors, and factor rotation was performed to minimize the correlation between factors. In order to determine which factors could be included to describe different dietary patterns, the evaluation was based on the eigenvalue (>1), the scree plot, the interpretability of the factors, and their professional significance. In this study, factors with absolute factor loadings > 0.3 [21] were retained as components of dietary patterns [22]. The scores of comprehensive factors were divided into quartiles, with Q1, Q2, Q3, and Q4 representing the scores from lowest to highest, respectively.

### 2.4. Statistical Analysis

The questionnaire was sorted and coded uniformly. Epidata version 3.1 was used for double data input to establish the original database. Data were described as n (%) for categorical variables and described as mean (95% CI) for continuous variables. Factor analysis was used to construct dietary patterns. Continuous variables were tested for normality. The Mann–Whitney U test was applied to compare them. Chi-square tests and the chi-square trend test were applied to compare categorical variables.

This study was cross-sectional, and the prevalence of anemia was greater than 10%. If we reported the odds ratio, it could have overestimated the association between anemia and the independent variables. In such cases, the prevalence ratio is the best measure of association, and therefore, robust Poisson regression analysis was fitted to identify predictors of anemia. The prevalence ratio (PR) and 95% CI were calculated, and linear trends of PRs were estimated. One unadjusted model and one covariate-adjusted model were fit for each dietary pattern. Model 1 was unadjusted. Based on the surveys and references, Model 2 was adjusted for additional factors, including parental education [23], gender, boarding status, alcohol consumption [24], BMI Z score [25], and growth retardation status.

Gender was selected as a stratification variable because the prevalence of anemia in girls was consistently higher than in boys [26]. Age was also selected as a stratification variable. The age criteria for stratified analysis were determined based on the onset of puberty boundary, which was set at 12.0 years for girls at menarche and 13.5 years for boys with testicular and pubic hair development as well as spermatorrhea [27,28]. SPSS version 26.0 was used for all computations, and a *p*-value (two-sided) < 0.05 was considered to be statistically significant. GraphPad Prism 8 version 8.0 and Microsoft Excel 2019 were used for charting.

## 3. Results

### 3.1. Participant Characteristics

Overall, 1476 children with complete data were included (53.3% male, n = 787; 46.6% female, n = 689) in this study. The age range of the children was between 9 and 16 years, with a mean age of 12.87 (12.81, 12.94) years, as shown in Table 2. The overall prevalence of anemia was 10.43%, with 6.1% in boys and 15.38% in girls. In the BMI Z score groups, the prevalence of anemia was highest in the underweight group (11.54%). Compared with non-anemic children, anemic children were more likely to be female, be underweight, and have an earlier sleeping time (*p* < 0.05).

### 3.2. Dietary Patterns

**Figure 2:**
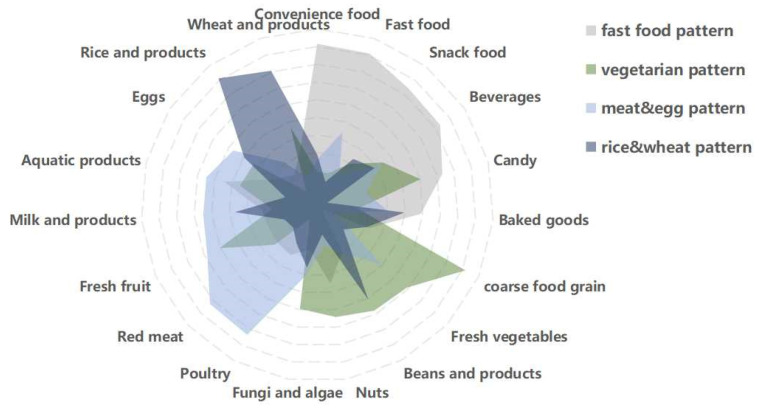
Radar chart of different dietary patterns obtained by factor analysis.

Dietary patterns, determined by principal component analysis, are shown in Figure 2 and Table 3. Four dietary patterns were generated and explained using factor loading and interpretability. Factor analysis identified 4 main dietary patterns from 19 food groups, which accounted for 13.52% (fast food pattern), 10.33% (vegetarian pattern), 9.99% (meat and egg pattern), and 7.43% (rice and wheat pattern) of variance, and together accounted for 41.26% of the total variance. After varimax rotation, the factor loading matrix of the food groups was obtained and is shown in Table 3. The fast food pattern was characterized by convenience foods, fast food, snacks, and beverages. The vegetarian pattern was characterized by whole grains, fresh vegetables, fruits, and soy products. The meat and egg pattern was characterized by poultry, livestock, seafood, fruits, and dairy products. The rice and wheat pattern was characterized by rice products, wheat products, and soy products.

### 3.3. Characteristics of Quartiles (Q) of Dietary Patterns in Study Participants

The characteristics of the Q1 (lowest) and Q4 (highest) quartiles of the four dietary patterns are shown in Table 4. The analysis found that children who tried smoking or drinking, had a late bedtime (≥21:00), and had shorter sleep duration were more likely to coincide with the fast food pattern. Girls, children with fathers with higher education levels, children engaged in high-intensity physical activity (≥3 times/week), children with longer sedentary time, children who reported going to bed earlier (<21:00), and children suffering from malnutrition were more likely to coincide with the vegetarian pattern. Children in the highest quartile of the meat and egg pattern were more likely to be older, male, live in a boarding school, have parents with higher education levels, have tried drinking, engage in high-intensity physical activity (≥3 times/week), and have longer sedentary time. Participants in the highest quartile of the rice and wheat pattern were more likely to be male, engage in high-intensity physical activity (≥3 times/week), and have longer sleep duration.

In terms of food intake, Q4 of the meat and egg pattern showed the highest intake of animal products (206 g/d), whereas other groups demonstrated an intake of ≤140 g/d. Q4 of the vegetarian pattern showed the highest intake of fresh vegetables (296 g/d), whereas other groups demonstrated an intake of ≤280 g/d. Q4 of the rice and wheat pattern showed the highest intake of rice and rice products (306 g/d), whereas other groups demonstrated an intake of ≤300 g/d.

### 3.4. Association Analysis between Dietary Patterns and Anemia

#### 3.4.1. Analysis of Dietary Patterns and Anemia

As shown in Table 5, the Mantel–Haenszel chi-square test results show that there was a linear relationship between the tendency level of the fast food pattern and the level of anemia, *p* < 0.05. The Pearson correlation result shows that R = 0.056, *p* < 0.05. This suggests that the higher the tendency towards the fast food pattern, the more severe the anemia.

Robust Poisson regression analysis showed that after adjusting for factors including age, gender, boarding status, parental education level, alcohol consumption, BMI Z score, and growth retardation, the high-tendency group (Q4) of the fast food pattern had a higher risk of anemia in children compared to the low-tendency group (Q1) (PR = 1.549, 95% CI: 1.002~2.396, *p* = 0.048).

#### 3.4.2. Robust Poisson Regression Analysis of Dietary Patterns and Anemia in Children of Different Genders and Ages

**Figure 3:**
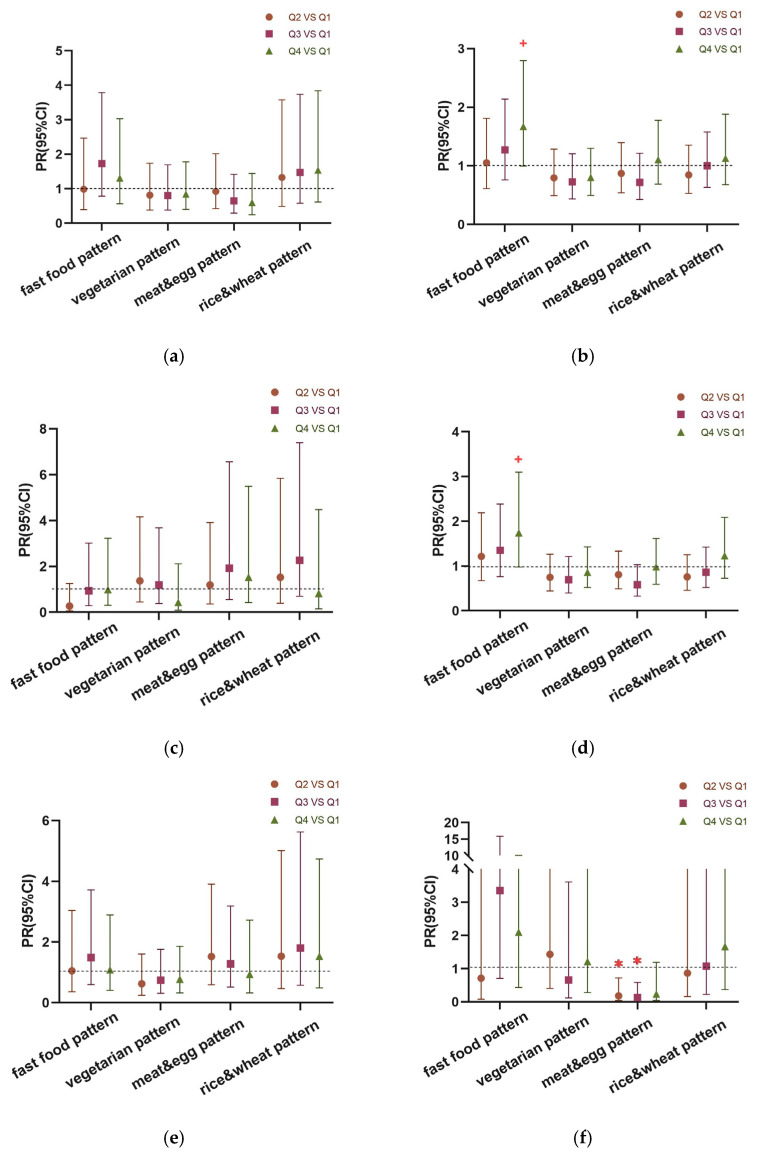

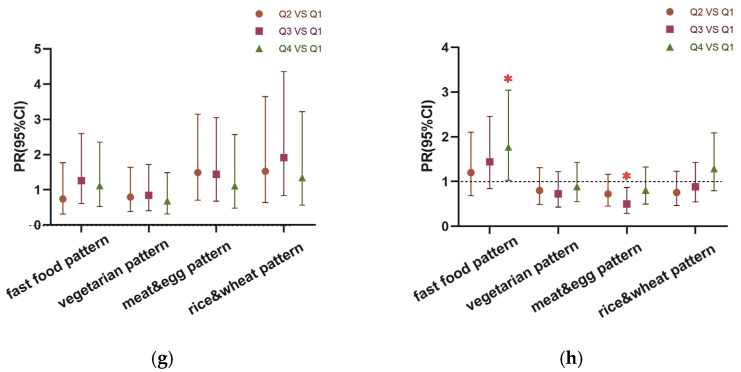
Robust Poisson regression analysis was used for analysis. Odds ratio and 95% CI in the second (Q2), third (Q3), and highest (Q4) groups of the four dietary patterns compared to the lowest group (Q1). (**a**) Males, n = 787; (**b**) females, n = 689; (**c**) females aged < 12 years, n = 158; (**d**) females aged ≥ 12 years, n = 531; (**e**) males aged < 13.5 years, n = 492; (**f**) males aged ≥ 13.5 years, n = 295; (**g**) males aged < 13.5 years and females aged < 12.0 years, n = 650; (**h**) males aged ≥ 13.5 years and females aged ≥ 12.0 years, n = 826. All models were adjusted for age, boarding status, parental education level, alcohol consumption, BMI Z score, and growth retardation. “✱” means *p*-value < 0.05; ”+”means *p*-value < 0.06.

The stratified analysis results are shown in Figure 3. After stratifying by gender, girls in the highest quartile (Q4) of the fast food pattern were more likely to be anemic than those in the lowest (Q1), with marginal significance (PR = 1.670, 95% CI: 0.997~2.797, *p* = 0.051) (Figure 3b). After stratifying by gender and age, girls aged ≥ 12.0 years in the highest quartile (Q4) of the fast food pattern were more likely to be anemic than those in the lowest (Q1), with marginal significance (PR = 1.740, 95% CI: 0.977~3.097, *p* = 0.059) (Figure 3d). Boys aged ≥ 13.5 years in the high quartile(Q3) of the meat and egg pattern were less likely to be anemic than those in the lowest (Q1) (PR = 0.237, 95% CI: 0.031~0.590, *p* = 0.007) (Figure 3f). Children entering puberty in the highest quartile (Q4) of the fast food pattern were more likely to be anemic than those in the lowest (Q1) (PR = 1.767, 95% CI: 1.026~3.043, *p* = 0.039), whereas those in the high quartile(Q3) of the meat and egg pattern were less likely to be anemic than those in the lowest quartile (Q1) (PR = 0.498, 95% CI: 0.286~0.866, *p* = 0.013) (Figure 3h).

## 4. Discussion

Anemia is a significant public health problem that threatens the health of billions of people worldwide. In 2019, the “Nutrition Improvement Plan for Rural Compulsory Education Students” in China found that the prevalence of anemia among children aged 6–17 in rural areas was 8.7%, with a higher prevalence among girls (10.0%) than boys (7.4%) [3]. The “National Student Physical Fitness and Health Survey” in 2014 found that the prevalence of anemia among children aged 7–14 years in Guangdong Province (located in south China; Guangzhou is its capital) was 10.6%, with a prevalence of 9.8% among boys and 11.5% among girls; the prevalence was 8.7% among urban children and 12.6% among rural children [29]. In this survey, the overall prevalence of anemia among the participants was 10.43%, with a prevalence of 6.1% among boys and 15.38% among girls. The prevalence of anemia in the girls of this study differed significantly from that of the “National Student Physical Fitness and Health Survey,” which may be related to the age composition of girls. In conclusion, the anemia status of local children deserves attention.

In this cross-sectional study, we identified four dietary patterns, including a fast food pattern, a vegetarian pattern, a meat and egg pattern, and a rice and wheat pattern. Dietary patterns can reflect children’s dietary intake more comprehensively than individual nutrients or foods [5,9,30]. The cumulative contribution rate of the fast food pattern was the highest (13.52%), which indicates that the existing diet of children is greatly influenced by Western culture [31]. Except for the fast food pattern, the other three dietary patterns reflected certain characteristics of Cantonese cuisine. Cantonese cuisine is a typical representative of the Eastern healthy dietary pattern, which is characterized by a rich variety of fresh ingredients, including various local fresh vegetables and fruits, poultry, and seafood. The dietary patterns examined in this study are of great significance for providing nutritional guidance to children with anemia.

The characteristic of the fast food pattern was a high intake of fast food, convenience food, beverages, and sweets, and it was positively associated with the level of anemia in our study. Anemia is closely related to nutrients such as iron [32], protein, vitamin B_12_, and folic acid [33,34]. About 80% of iron in the body is related to the production of hemoglobin in red blood cells [35,36]; thus, iron deficiency is regarded as the most common cause of anemia. A study in Poland showed that fast food can only cover 4% of the recommended intake of iron [37]. After stratifying for gender, a correlation analysis found that the fast food pattern was positively correlated with anemia in girls, especially in girls after menarche, with marginal significance. A study in Greece found that girls who consumd a large amount of fast food were more likely to have iron deficiency [38]. Most fast food is characterized by high fat, salt, and sugar. Long-term high-fat diets decrease hepatic iron storage [39]. Beverages and candy in the fast food pattern also had negative effects on iron metabolism. One study observed that ferritin was significantly decreased in subjects consuming sugar-containing beverages [40]. An experiment on rats showed that a high-fructose diet can induce systemic iron deficiency, likely due to the activation of inflammation. Hepcidin upregulation induced by NF-κB and JAK2/STAT3 activation reduces iron intake. Hepcidin upregulation decreased intestinal iron absorption and sequestration of the metal in macrophages and hepatocytes, leading to systemic iron deficiency [41]. Therefore, children should be guided to reduce their intake of fast food, beverages, and candy during nutritional intervention. The marginal significance may be due to the limited sample size, so future studies will increase the sample size to explore the association between the two.

In our study, the association analysis was carried out after age and gender stratification. The results show that the meat and egg pattern was negatively associated with anemia among boys after spermatorrhea. The utilization rate of heme iron in animal food is relatively high [42] because heme forms soluble complexes with other dietary components in the gut lumen. Soluble complexes are readily absorbed by the body. Iron is then released from heme after it enters intestinal enterocytes [43]. The meat and egg pattern was characterized by poultry and livestock, which can effectively increase iron absorption and intake. In addition, the meat and egg pattern can provide abundant vitamins, and its intake of fresh fruits was the highest of the four patterns. Guangdong is rich in fruits, mainly including tropical fruits such as guava, longan, and litchi, due to its special climate. One study showed that the risk of low serum iron levels in girls who did not consume guava was increased by 3.8 times [44]. Vitamin C can change the expression of iron-regulating RNA in HepG2 cells [45], and dietary interventions combining iron and vitamin C are more effective in improving iron status [44,46]. A study in South Africa showed that vitamins A and B_12_ have a protective effect on anemia in children aged 5–12 years [47]. The children in this study can achieve a balance of adequate vitamin and iron intake through the meat and egg pattern.

Finally, when the boys and girls entering puberty were combined for analysis of their dietary patterns and anemia, it was found that the fast food pattern was positively correlated with anemia and that the meat and egg pattern was negatively correlated with anemia in children entering puberty. These associations were not observed in prepubescent children. Studies have shown that the iron protein in pubescent boys is lower than that in prepubescent boys [48]. Total daily iron requirements in girls almost doubles during puberty, ranging from 1.22–1.46 mg/day before menarche to 1.39–2.54 mg/day after menarche [38]. The demand for iron in pubescent children increases, so the harmful effects of the fast food pattern and the protective effects of the meat and egg pattern became more apparent after children had entered puberty. This provides direction for future nutritional interventions and suggests that the iron intake in the diet needs to be increased for children entering puberty.

Although the vegetarian and rice and wheat patterns were not found to be associated with anemia, there have been related studies on the foods included in these patterns. The absorption rate of non-heme iron in plant-based foods is lower than that in animal-based foods, and phytate and tannins in plants can chelate non-heme iron [49]. Vegetarians are more likely to develop iron-deficiency anemia [50]. Studies in China [51] and Japan [11] have both shown that a rice-based diet has a harmful effect on anemia. However, a study in India showed that increased consumption of coarse cereals could reduce anemia prevalence in Indian women. None of these studies were conducted on children aged 9–16 years, so further studies with larger sample sizes are needed to investigate the effects of these two dietary patterns on anemia.

This study also found that children who tried smoking and drinking and stayed up late tended to adopt the fast food pattern. The results indicate that teenagers who tended towards unhealthy diets also engaged in unhealthy daily behaviors, which is consistent with the findings of Lidia Wadolowska’s research [52]. Children who lacked sleep tended to prefer the fast food pattern. Research has shown that lack of sleep may affect appetite-regulating hormones such as leptin, increase hunger, and lead to more frequent consumption of fast food, and excessive intake of fast food can lead to sleep disorders and form a vicious cycle [53].At the same time, we also observed that compared to non-anemic children, anemic children tended to go to bed earlier. This may be due to the fatigue experienced by anemic patients [54], leading to early sleep. The specific causal relationship needs further in-depth research. It is worth noting that many underweight children had a diet that was closer to the vegetarian pattern [50], mainly because there was too little meat in the vegetarian pattern. A lack of meat in the diet can cause children to lack nutrients such as iron, zinc, calcium, and vitamin B_12_ [49], which are involved in all aspects of children’s growth and development [55], and lead to thinness. Compared to girls who tended towards the vegetarian pattern, boys tended to prefer the meat and egg pattern. This is consistent with a study in the UK, where male students tended to buy convenience foods, red meat, and alcohol [56].

Firstly, regarding the strengths of the study, a large number of studies focus on the problem of excess nutrition in children and adolescents, such as overweight and obesity, at present. This paper focuses on anemia in children and adolescents in rural areas. The results show that anemia in this population should be paid enough attention. Secondly, this paper explores the influencing factors of anemia in children and adolescents from the perspective of dietary patterns and avoids the monogeneity of single-nutrient research. Our results can provide a scientific basis for improvement measures. However, the present investigation has some shortcomings. Firstly, the dietary data reflect children’s long-term dietary habits based on a survey of their diet over the previous month. However, due to the limited knowledge of children about food, recall bias could not be avoided in the survey. Secondly, due to the differences in dietary cultures across different regions, caution is required when extrapolating the study results. Thirdly, this is a cross-sectional study, and it cannot reveal the causal relationship between dietary patterns and anemia. It is recommended to conduct prospective cohort studies to explore the causal relationship between dietary patterns and anemia.

## 5. Conclusions

In summary, the prevalence of anemia in rural children in Guangzhou remains a significant concern. This study identified four dietary patterns, including a fast food pattern, a vegetarian pattern, a meat and egg pattern, and a rice and wheat pattern. The study shows that different dietary patterns play different roles in anemia among the children. The fast food pattern was a risk factor for anemia in children, especially in girls after menarche. The meat and egg pattern was a protective factor for anemia in children entering puberty, especially in boys after spermatorrhea. These findings can be used to develop nutritional interventions for the prevention of nutrition-related anemia in children.

## Figures and Tables

**Table 1:** Food groups used in the factor analysis.

Number	Food Group	Examples of Food Items
1	Rice and rice products	Rice, rice gruel, rice noodles
2	Wheat and wheat products	Wheat flour noodles, wheat buns, dumplings Fried bread, fried breadsticks
3	Coarse food grain	Corn, cornmeal Potato, sweet potato,
4	Beans and bean products	Soybean, soybean milk, tofu, bean curd, dried bean curd
5	Fresh vegetables	Cabbage, tomato, lettuce
6	Mushrooms and algae	Mushroom, laver, kelp
7	Fresh fruits	Banana, apple, berries
8	Milk and dairy products	Milk, milk powder, yogurt, cheese
9	Red meat	Pork, beef, goat, lamb Liver, kidney, large intestine Ham sausage, bacon
10	Poultry	Chicken, duck, goose
11	Aquatic products	Fish, shrimp, crab
12	Eggs	Eggs
13	Nuts	Peanuts, almonds, walnuts, hazelnuts
14	Baked goods	Cookies, cakes, bread
15	Candy	Sugar, jam, jelly, candies, chocolate, candied fruit
16	Snack food	Spicy strips, fried puffed snacks
17	Convenience food	Instant noodles, instant rice noodles
18	Fast food	Hamburger, fried chicken
19	Beverages	Carbonated drinks, prepackaged juice, milk beverages, sweet tea beverages, vegetable protein drinks, sports beverages

**Table 2:** Demographic and lifestyle characteristics of the study participants.

Variable	Total	Anemia		*p*
		Yes	No	
Age, mean (95% CI)	12.87 (12.81, 12.94)	12.99 (12.78, 13.20)	12.86 (12.79, 12.93)	0.409
Sex, n (%)				
Male	787	48 (6.10)	739 (93.90)	**<0.001**
Female	689	106 (15.38)	583 (84.62)	
Education of father, n (%)				
Primary or below	66	10 (15.15)	56 (84.85)	0.657
Middle school	722	69 (9.56)	653 (90.44)	
Senior high school	355	38 (10.70)	317 (89.30)	
Junior college or above	245	25 (10.20)	220 (89.80)	
Unknown	88	12 (13.64)	76 (86.36)	
Education of mother, n (%)				
Primary or below	116	12 (10.34)	104 (89.66)	0.159
Middle school	724	83 (11.46)	641 (88.54)	
Senior high school	302	32 (10.60)	270 (89.40)	
Junior college or above	247	20 (8.10)	227 (91.90)	
Unknown	87	7 (8.05)	80 (91.95)	
Boarding, n (%)				
Yes	594	66 (11.11)	528 (88.89)	0.485
No	882	88 (9.98)	794 (90.02)	
Tried smoking, n (%)				
Yes	133	13 (9.77)	120 (90.23)	0.794
No	1343	141 (10.50)	1202 (89.50)	
Alcohol consumption, n (%)				
Yes	309	32 (10.36)	277 (89.64)	0.960
No	1167	122 (10.45)	1045 (89.55)	
Moderate-to-high-intensity exercise, n (%)				
<3 times/week	264	32 (12.12)	232 (87.88)	0.322
≥3 times/week	1212	122 (10.07)	1090 (89.93)	
Sedentary time, mean (95% CI)	8.07 (7.97, 8.18)	8.12 (7.78, 8.46)	8.07 (7.96, 8.18)	0.692
Bedtime, n (%)				
<9 p.m.	59	15 (25.42)	44 (74.58)	**<0.001**
≥9 p.m.	1417	139 (9.81)	1278 (90.19)	
Sleep time, mean (95% CI)	8.95 (8.88, 9.02)	8.84 (8.63, 9.06)	8.96 (8.89, 9.04)	0.322
BMI Z score, n (%)				
Underweight	78	9 (11.54)	69 (88.46)	**0.032**
Normal weight	1168	128 (10.96)	1040 (89.04)	
Overweight	171	17 (9.94)	154 (90.06)	
Obesity	59	0 (0.00)	59 (100.00)	
Growth retardation, n (%)				0.631
Yes	15	1 (6.67)	14 (93.30)	
No	1461	153 (10.47)	1308 (89.53)	

Note: Data are shown as number of cases (%) for categorical variables and mean (95% CI) for continuous variables. The Mann–Whitney U test and the chi-square trend test were used for analysis. The bold *p*-value means “<0.05”.

**Table 3:** Factor loadings and dietary patterns for 19 food groups obtained by factor analysis.

Food	Fast Food Pattern	Vegetarian Pattern	Meat and Egg Pattern	Rice and Wheat Pattern
Convenience food	0.716			
Fast food	0.710			
Snack food	0.641			
Beverages	0.632			
Candy	0.532	0.403		
Baked goods	0.389			
Coarse food grain		0.718		
Fresh vegetables		0.489		
Beans and bean products		0.479		0.408
Nuts		0.442		
Mushrooms and algae		0.395		
Poultry			0.635	
Red meat			0.626	
Fresh fruits		0.406	0.482	
Milk and dairy products			0.449	
Aquatic products	0.344		0.449	
Poultry			0.368	
Rice and rice products				0.712
Wheat and wheat products				0.607

Note: PCA (principal component analysis) was used for analysis. Factor loadings of <0.3 in absolute terms were excluded for simplicity.

**Table 4:** Quartile (Q) characteristics of dietary pattern scores in the study participants.

Variable	Fast Food Pattern			Vegetarian Pattern			Meat and Egg Pattern			Rice and Wheat Pattern		
	Q1	Q4	*p*	Q1	Q4	*p*	Q1	Q4	*p*	Q1	Q4	*p*
Age, mean (95% CI)	12.91 (12.77, 13.05)	12.85 (12.72, 12.99)	0.528	12.89 (12.75, 13.03)	12.84 (12.71, 12.97)	0.380	12.57 (12.43, 12.72)	13.03 (12.9, 13.15)	**<0.001**	12.85 (12.71, 12.99)	12.88 (12.75, 13.02)	0.522
Gender, n (%)												
Male	197 (48.28)	211 (51.72)	0.300	221 (54.17)	187 (45.83)	**0.012**	166 (42.35)	226 (57.65)	**<0.001**	134 (33.33)	268 (66.67)	**<0.001**
Female	172 (52.12)	158 (47.88)		148 (44.85)	182 (55.15)		203 (58.67)	143 (41.33)		235 (69.94)	101 (30.06)	
Boarding, n (%)												
Yes	148 (47.13)	166 (52.87)	0.180	144 (50.17)	143 (49.83)	0.940	113 (39.79)	171 (60.21)	**<0.001**	136 (48.92)	142 (51.08)	0.649
No	221 (52.12)	203 (47.88)		225 (49.89)	226 (50.11)		256 (56.39)	198 (43.61)		233 (50.65)	227 (49.35)	
Education of father, n (%)												
Primary or below	16 (50.00)	16 (50.00)	0.125	15 (51.72)	14 (48.28)	**0.042**	14 (51.85)	13 (48.15)	**<0.001**	15 (55.56)	12 (44.44)	**0.037**
Middle school	173 (48.19)	186 (51.81)		200 (54.35)	168 (45.65)		218 (58.45)	155 (41.55)		168 (46.54)	193 (53.46)	
Senior high school	93 (51.38)	88 (48.62)		70 (40.00)	105 (60.00)		73 (42.69)	98 (57.31)		91 (51.41)	86 (48.59)	
Junior college or above	71 (58.20)	51 (41.80)		63 (50.81)	61 (49.19)		40 (34.48)	76 (65.52)		65 (49.62)	66 (50.38)	
Unknown	16 (36.36)	28 (63.64)		21 (50.00)	21 (50.00)		24 (47.06)	27 (52.94)		30 (71.43)	12 (28.57)	
Education of mother, n (%)												
Primary or below	30 (52.63)	27 (47.37)	0.067	28 (57.14)	21 (42.86)	0.845	35 (64.81)	19 (35.19)	**<0.001**	20 (42.55)	27 (57.45)	0.159
Middle school	177 (49.44)	181 (50.56)		180 (49.86)	181 (50.14)		202 (56.74)	154 (43.26)		182 (49.46)	186 (50.54)	
Senior high school	74 (48.68)	78 (51.32)		75 (49.34)	77 (50.66)		63 (42.86)	84 (57.14)		82 (55.03)	67 (44.97)	
Junior college or above	74 (57.81)	54 (42.19)		65 (47.79)	71 (52.21)		45 (35.16)	83 (64.84)		60 (44.78)	74 (55.22)	
Unknown	14 (32.56)	29 (67.44)		21 (52.50)	19 (47.50)		24 (45.28)	29 (54.72)		25 (62.50)	15 (37.50)	
Alcohol consumption, n (%)												
Yes	57 (34.76)	107 (65.24)	**<0.001**	75 (49.34)	77 (50.66)	0.856	68 (41.72)	95 (58.28)	**0.017**	82 (50.00)	82 (50.00)	1.000
No	312 (54.36)	262 (45.64)		294 (50.17)	292 (49.83)		301 (52.35)	274 (47.65)		287 (50.00)	287 (50.00)	
Tried smoking, n (%)												
Yes	15 (22.06)	53 (77.94)	**<0.001**	41 (57.75)	30 (42.25)	0.169	31 (49.21)	32 (50.79)	0.895	31 (42.47)	42 (57.53)	0.174
No	354 (52.84)	316 (47.16)		328 (49.18)	339 (50.82)		338 (50.07)	337 (49.93)		338 (50.83)	327 (49.17)	
Sleep time, mean (95% CI)	9.04 (8.91, 9.17)	8.91 (9.17, 9.05)	**0.003**	8.88 (8.75, 9.02)	9.04 (8.89, 9.19)	0.057	9.03 (8.88, 9.18)	8.98 (8.84, 9.12)	0.373	8.80 (8.65, 8.95)	9.07 (8.94, 9.20)	**0.015**
Moderate-to-high physical activity, n (%)												
<3 times/week	55 (47.41)	61 (52.59)	0.544	83 (58.45)	59 (41.55)	**0.025**	85 (64.89)	46 (35.11)	**<0.001**	90 (61.64)	56 (38.36)	**0.002**
≥3 times/week	314 (50.48)	308 (49.52)		286 (47.99)	310 (52.01)		284 (46.79)	323 (53.21)		279 (47.13)	313 (52.87)	
Sedentary time, mean (95% CI)	8.12(7.91, 8.33)	8.12(7.91, 8.34)	0.897	7.88(7.68, 8.09)	8.27(8.06, 8.49)	**0.006**	7.69(7.49, 7.9)	8.28(8.08, 8.48)	**<0.001**	8.04(7.84, 8.23)	8.01(7.79, 8.22)	0.306
Bedtime, n (%)												
<9 p.m.	22 (73.33)	8 (26.67)	**0.008**	9 (31.03)	20 (68.97)	**0.035**	13 (43.33)	17 (56.67)	0.455	15 (45.45)	18 (54.55)	0.593
≥9 p.m.	347 (49.01)	361 (50.99)		360 (50.78)	349 (49.22)		356 (50.28)	352 (49.72)		354 (50.21)	351 (49.79)	
Malnutrition, n (%)												
Yes	18 (48.65)	19 (51.35)	0.866	11 (31.43)	24 (68.57)	**0.023**	12 (42.86)	16 (57.14)	0.441	15 (44.12)	19 (55.88)	0.482
No	351 (50.07)	350 (49.93)		358 (50.92)	345 (49.08)		357 (50.28)	353 (49.72)		354 (50.28)	350 (49.72)	
Overweight/obesity, n (%)												
Yes	61 (51.26)	58 (48.74)	0.764	58 (49.15)	60 (50.85)	0.841	52 (47.27)	58 (52.73)	0.535	48 (41.74)	67 (58.26)	0.054
No	308 (49.76)	311 (50.24)		311 (50.16)	309 (49.84)		317 (50.48)	311 (49.52)		321 (51.52)	302 (48.48)	
Growth retardation, n (%)												
Yes	4 (50.00)	4 (50.00)	1.000	3 (50.00)	3 (50.00)	1.000	4 (57.14)	3 (42.86)	0.704	2 (25.00)	6 (75.00)	0.136
No	365 (50.00)	365 (50.00)		366 (50.00)	366 (50.00)		365 (49.93)	366 (50.07)		367 (50.27)	363 (49.73)	
Food intake (g/d), mean ^a^												
Animal foods	107	137	**<0.001**	108	133	**<0.001**	47	206	**<0.001**	114	119	**0.016**
Rice and rice products	220	201	**0.012**	231	195	**<0.001**	187	215	**<0.001**	122	306	**<0.001**
Wheat and wheat products	58	66	**0.038**	40	78	**<0.001**	63	58	0.486	27	102	**<0.001**
Coarse food grain	35	38	0.186	9	71	**<0.001**	39	30	0.769	25	40	**<0.001**
Fresh vegetables	281	169	**<0.001**	101	296	**<0.001**	129	252	**<0.001**	197	199	**0.017**
Fresh fruits	213	206	0.292	112	272	**<0.001**	113	284	**<0.001**	201	190	0.782
Milk and dairy products	264	295	**0.008**	266	288	**0.035**	149	379	**<0.001**	215	321	**<0.001**
Eggs	43	39	**0.020**	29	50	**<0.001**	19	54	**<0.001**	24	52	**<0.001**
Beans and bean products	14	17	0.241	7	24	**<0.001**	12	16	**<0.001**	7	22	**<0.001**

Note: Data are shown as number of cases (%) for categorical variables and mean (95% CI) for continuous variables. The chi-square trend test, the chi-square test, and the Mann–Whitney U test were used for analysis. a The 95% CIs of food intake are in the Supplementary Materials because they are estimated values. The bold p-value means “<0.05”.

**Table 5:** Analysis of the association between dietary patterns and anemia.

Dietary Pattern	Non-Anemia	Anemia	*p*	Model 1		Model 2	
				PR (95% CI)	*p*	PR (95% CI)	*p*
Fast food pattern, n (%)							
Q1	338 (91.60)	31 (8.40)	**0.033 ^a^**	1		1	
Q2	336 (91.06)	33 (8.94)		1.064 (0.666, 1.700)	0.793	1.045 (0.656, 1.663)	0.851
Q3	325 (88.08)	44 (11.92)		1.419 (0.917, 2.195)	0.115	1.382 (0.898, 2.129)	0.141
Q4	323 (87.53)	46 (12.47)		1.483 (0.963, 2.285)	0.073	1.549 (1.002, 2.396)	**0.048**
Vegetarian pattern, n (%)							
Q1	326 (88.35)	43 (11.65)	0.493	1		1	
Q2	331 (89.70)	38 (10.30)		0.883 (0.585, 1.334)	0.556	0.803 (0.535, 1.207)	0.293
Q3	334 (90.51)	35 (9.49)		0.813 (0.533, 1.241)	0.339	0.750 (0.491, 1.145)	0.183
Q4	331 (89.70)	38 (10.30)		0.883 (0.585, 1.334)	0.556	0.816 (0.544, 1.226)	0.329
Meat and egg pattern, n (%)							
Q1	323 (87.53)	46 (12.47)	0.128	1		1	
Q2	328 (88.89)	41 (11.11)		0.891 (0.600, 1.323)	0.568	0.906 (0.607, 1.353)	0.631
Q3	338 (91.60)	31 (8.40)		0.673 (0.437, 1.038)	0.073	0.710 (0.456, 1.106)	0.130
Q4	333 (90.24)	36 (9.76)		0.782 (0.518, 1.181)	0.243	0.886 (0.578, 1.358)	0.580
Rice and wheat pattern, n (%)							
Q1	327 (88.62)	42 (11.38)	0.594	1		1	
Q2	333 (90.24)	36 (9.76)		0.857 (0.562, 1.306)	0.473	0.927 (0.611, 1.406)	0.722
Q3	329 (89.16)	40 (10.84)		0.952 (0.633, 1.432)	0.814	1.104 (0.741, 1.646)	0.625
Q4	333 (90.24)	36 (9.76)		0.857 (0.562, 1.306)	0.473	1.201 (0.787, 1.834)	0.394

Note: The chi-square trend test and robust Poisson regression analysis were used for analysis. Q1 was the reference group. Model 1: unadjusted; Model 2: adjusted for age, gender, boarding status, parental education level, alcohol consumption, BMI Z score, and growth retardation. ^a^ Pearson’s R = 0.056, *p* = 0.033 (a detailed table is in the Supplementary Materials). The bold p-value means “<0.05”.

## Data Availability

The data are not publicly available due to privacy. The data presented in the analyses for this study are available on request from the corresponding author.

## Supplementary Materials

The following supporting information can be downloaded at: https://www.mdpi.com/article/10.3390/nu15194133/s1. Table S1: Description of Robust Poisson regression assignment; Table S2. Symmetric Measures; Table S3: Detailed dietary intake.
