# Supplementary material for: Dietary Patterns and Association with Anemia in Children Aged 9–16 Years in Guangzhou, China: A Cross-Sectional Study
Source: Nutrients. 2023 Sep 25;15(19):4133. doi: 10.3390/nu15194133 (PMC10574347; doi:10.3390/nu15194133)
Supplement: Supplementary file 1 [file nutrients-15-04133-s001.zip › nutrients-2589643-supplementary.pdf]

Supplementary Materials:

Table S1: Description of Robust Poisson regression assignment

Factors	Variable	Explain
Anemia	Y	non-anemia=0;anemia=1;
Dietary pattern quartiles group	X1	Q2=1；Q3=2；Q4=3；Q1=4；
Age	X2	Continuous variable
Gender	X3	Male=1；Female=2
Boarding status	X4	Yes=1；No=2；
Father education level	X5	Primary school and below=1;Junior high school=2; high school technical school=3;junior college and above=4; ignorant=5
Mother education level	X6	Primary school and below=1;Junior high school=2; high school technical school=3;junior college and above=4; ignorant=5
Trying alcohol	X7	Yes=1；no=2；
BMI Z score	X8	Underweight=1;Normal weight=2;Overweight=3;Obesity=4
Growth retardation	X9	Yes=1；no=2；

Table S2:Symmetric Measures

		Value	Asymptotic Standard Error[a]	Approximate T[b]	Approximate Significance
Interval by Interval	Pearson's R	.056	0.026	2.134	0.033[c]
Ordinal by Ordinal	Spearman Correlation	.056	0.026	2.134	0.033[c]
N of Valid Cases		1476			

Note:a. Not assuming the null hypothesis.b. Using the asymptotic standard error assuming the null hypothesis.c. Based on normal approximation.

Table S3:Detailed dietary intake

Variable	fast food pattern			vegetarian pattern			meat & egg pattern			rice & wheat pattern		
	**Q1**	**Q4**	***p***	**Q1**	**Q4**	***p***	**Q1**	**Q4**	***p***	**Q1**	**Q4**	***p***
**Food intake (g/d), Mean(95%CI)**												
Animal foods	107.16 (96.85,117.48)	136.6 (123.88,149.33)	**<0.001**	107.83 (96.84,118.82)	132.66 (121.66,143.66)	**<0.001**	46.57 (42.94,50.19)	205.54 (192.2,218.88)	**<0.001**	114.23 (101.24,127.22)	118.73 (108.32,129.15)	**0.016**
Rice and rice products	219.59 (209.44,229.75)	201.05 (190.88,211.21)	**0.012**	231.45 (219.98,242.92)	195.44 (186.22,204.67)	**<0.001**	187.22 (177.33,197.1)	214.87 (203.91,225.82)	**<0.001**	122.35 (117.02,127.69)	305.75 (294.88,316.63)	**<0.001**
Wheat and wheat products	58.43 (52.14,64.71)	65.68 (58.95,72.4)	**0.038**	40.43 (35.68,45.18)	77.51 (70.73,84.29)	**<0.001**	62.75 (56.23,69.28)	57.83 (51.14,64.51)	0.486	26.5 (24.12,28.87)	102.41 (94.04,110.77)	**<0.001**
Coarse food grain	34.51 (30.13,38.9)	38.02 (30.79,45.25)	0.186	9.35 (8.06,10.65)	71.47 (63.48,79.46)	**<0.001**	38.68 (31.33,46.04)	30.18 (25.92,34.45)	0.769	25.39 (19.85,30.94)	39.97 (34.14,45.8)	**<0.001**
Fresh vegetables	280.57 (262.75,298.39)	168.54 (155.05,182.03)	**<0.001**	100.85 (93.36,108.35)	295.78 (276.63,314.93)	**<0.001**	128.85 (116.95,140.74)	252.15 (234.19,270.11)	**<0.001**	196.83 (179.19,214.47)	199.15 (185.61,212.7)	**0.017**
Fresh fruits	213.35 (195.07,231.62)	206.15 (187.32,224.98)	0.292	112.08 (102.18,121.99)	272.22 (252.16,292.29)	**<0.001**	112.61 (102.78,122.44)	283.9 (261.3,306.49)	**<0.001**	200.69 (181.41,219.96)	189.91 (174.84,204.99)	0.782
Milk and dairy products	263.51 (244.32,282.7)	294.81 (274.47,315.14)	**0.008**	265.93 (246.72,285.14)	287.73 (269.9,305.56)	**0.035**	149.26 (137.44,161.08)	379.01 (357.26,400.76)	**<0.001**	215.46 (199.84,231.07)	321.23 (299.14,343.31)	**<0.001**
Eggs	42.88 (38.82,46.93)	38.67 (34.73,42.61)	**0.020**	29.04 (25.97,32.12)	50.37 (45.86,54.88)	**<0.001**	19.41 (17.49,21.33)	53.5 (48.64,58.35)	**<0.001**	24.41 (21.99,26.83)	51.52 (46.85,56.19)	**<0.001**
Beans and bean products	14.44 (12.84,16.04)	16.57 (14.4,18.73)	0.241	6.82 (5.95,7.7)	24.27 (21.75,26.78)	**<0.001**	11.89 (9.81,13.96)	16.34 (14.53,18.14)	**<0.001**	6.85 (6.04,7.66)	22.01 (19.52,24.51)	**<0.001**
